# Supplementary figures and images for: Antigenic evolution of SARS-CoV-2 in immunocompromised hosts
Source: Evol Med Public Health. 2022 Nov 11;11(1):90–100. doi: 10.1093/emph/eoac037 (PMC10061940; doi:10.1093/emph/eoac037)

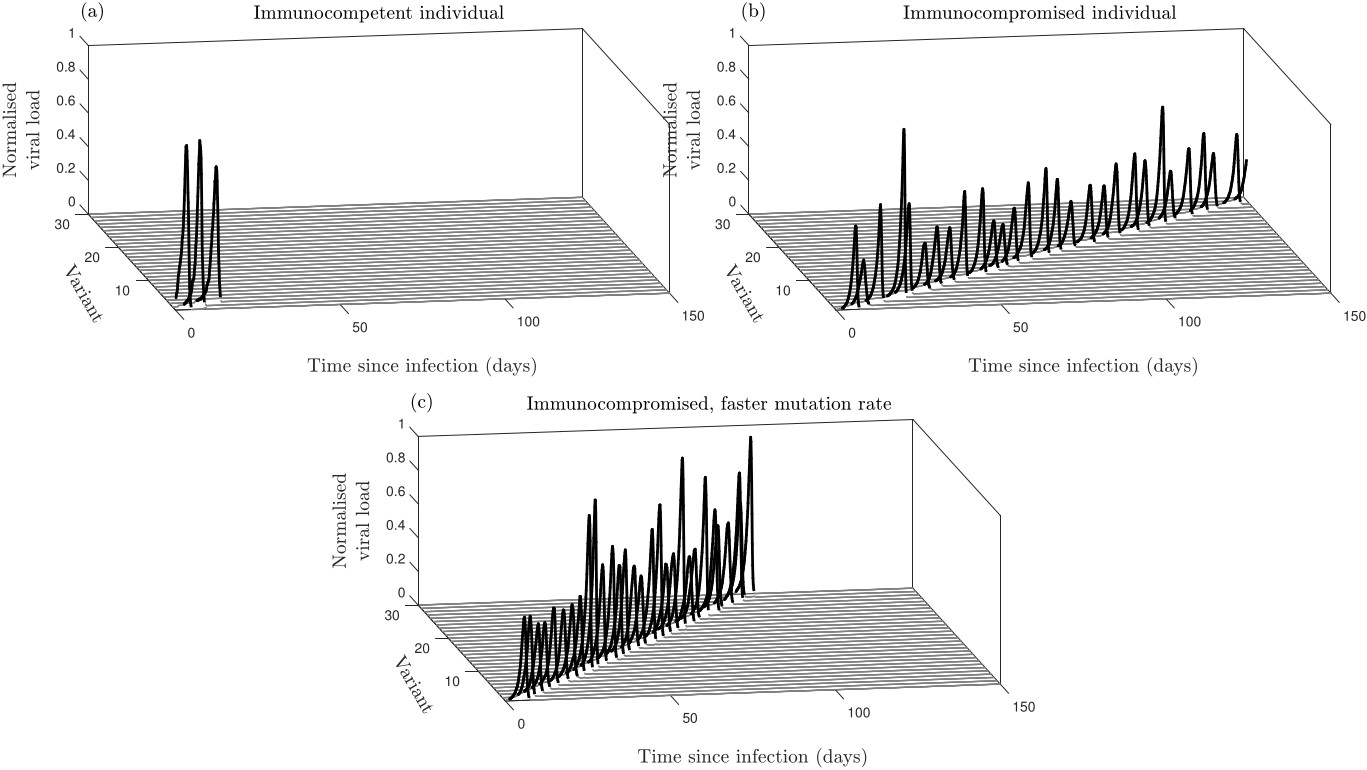

Supplement: eoac037_suppl_Supplementary_Figure_S1 [file eoac037_suppl_supplementary_figure_s1.jpeg]
