# Supplementary material for: Antigenic evolution of SARS-CoV-2 in immunocompromised hosts
Source: Evol Med Public Health. 2022 Nov 11;11(1):90–100. doi: 10.1093/emph/eoac037 (PMC10061940; doi:10.1093/emph/eoac037)
Supplement: eoac037_suppl_Supplementary_Table_S1 [file eoac037_suppl_supplementary_table_s1.docx]

| **Event** | **Rate** |
| --- | --- |
| Infection of host type $k\in\{H,C\}$ by variant $i$ | $\alpha_{\text{Inf, }k}^{i}= \beta_{i}S_{k}^{i}\left( I_{H}^{i}+I_{S}^{i} \right)$ |
| Gain of full immunity to variant $i$ due to infection by variant $j$ (host type $k\in\{H,C\}$) | $\alpha_{\text{Imm, }j\text{, }k}^{i}= \beta_{j}\sigma_{ij}S_{k}^{i}\left( I_{H}^{j}+I_{S}^{j} \right)$ |
| Recovery by host type $k\in\{H,C\}$ from variant $i$ | $\alpha_{\text{Rec, }k}^{i}= \gamma_{k}I_{k}^{i}$ |
| Mutation from variant $i$ to variant $i\pm1$ for host type $k\in\{H,C\}$. | $\alpha_{\text{Mut, }k}^{i}=\frac{\mu_{k}}{1+\delta_{i}}I_{k}^{i}$ |
