# Supplementary material for: Antigenic evolution of SARS-CoV-2 in immunocompromised hosts
Source: Evol Med Public Health. 2022 Nov 11;11(1):90–100. doi: 10.1093/emph/eoac037 (PMC10061940; doi:10.1093/emph/eoac037)
Supplement: eoac037_suppl_Supplementary_Table_S2 [file eoac037_suppl_supplementary_table_s2.docx]

| **Event** | **Rate** |
| --- | --- |
| Growth of variant $i$ | $\alpha_{\text{Grow}}^{i}= rV_{i}$ |
| Removal of variant $i$ by the corresponding immune response | $\alpha_{\text{Rem}}^{i}=\kappa V_{i}R_{i}$ |
| Removal of variant $i$ by immune response with index $j$ | $\alpha_{\text{Cross-rem,} j}^{i}=\kappa\tilde{\sigma}_{ij}V_{i}R_{j}$ |
| Mutation from variant $i$ to variant $i\pm1$ | $\alpha_{\text{Mut,}}^{i}=\frac{\tilde{\mu}}{1+\delta_{i}}V_{i}$ |
| Decay of immune response for variant $i$ | $\alpha_{\text{Dec}}^{i}=dR_{i}$ |
