# Supplementary material for: Antigenic evolution of SARS-CoV-2 in immunocompromised hosts
Source: Evol Med Public Health. 2022 Nov 11;11(1):90–100. doi: 10.1093/emph/eoac037 (PMC10061940; doi:10.1093/emph/eoac037)
Supplement: eoac037_suppl_Supplementary_Table_S3 [file eoac037_suppl_supplementary_table_s3.docx]

| **Parameter** | **Description** | **Value** |
| --- | --- | --- |
| $p$ | Proportion immunocompromised | 0.05 |
| $\xi$ | Strength of epistasis (proportional reduction in transmission) | 0.8 |
| $N$ | Population size | ${10}^{7}$ |
| $n$ | Number of variants | 30 |
| $\mu_{k}$ | Mutation rate for $k\in\{H,C\}$ | $0.01$ per day |
| $R_{0}$ | Basic reproductive number | 3.0 |
| $\gamma_{H}$ | Recovery rate (immunocompetent) | $1/7$ per day |
| $\gamma_{C}$ | Recovery rate (immunocompromised) | $1/140$ per day |
| $t_{max}$ | Final time | 1460 days |
| $\eta$ | Strength of cross-immunity | 10.0 |
