# Supplementary material for: Antigenic evolution of SARS-CoV-2 in immunocompromised hosts
Source: Evol Med Public Health. 2022 Nov 11;11(1):90–100. doi: 10.1093/emph/eoac037 (PMC10061940; doi:10.1093/emph/eoac037)
Supplement: eoac037_suppl_Supplementary_Table_S4 [file eoac037_suppl_supplementary_table_s4.docx]

| **Parameter** | **Description** | **Value** |
| --- | --- | --- |
| $n$ | Number of variants | 30 |
| $\tilde{\mu}$ | Mutation rate | $5\times{10}^{-3}$ per day |
| $r$ | Viral growth rate | 1.0 per day |
| $\kappa$ | Viral clearance rate by immune response | ${2\times10}^{-2}$ per unit viral load per day |
| $q$ | Relative strength of host immune system | 0.1 |
| $t_{max}$ | Final time | 150 days |
| $d$ | Decay rate of immune response | ${10}^{-4}$ per day |
| $\tilde{\eta}$ | Strength of cross-immunity | 0.1 |
