# Supplementary material for: Antigenic evolution of SARS-CoV-2 in immunocompromised hosts
Source: Evol Med Public Health. 2022 Nov 11;11(1):90–100. doi: 10.1093/emph/eoac037 (PMC10061940; doi:10.1093/emph/eoac037)
Supplement: eoac037_suppl_Supplementary_Legends [file eoac037_suppl_supplementary_legends.docx]

**Supplementary Figure 1**- Within-host dynamics. (a) Immunocompetent host (q=1.0), (b) Immunocompromised host (q=0.1), (c) immunocompromised host with faster mutation q=0.1,μ ̃=0.02). Values of the viral load are normalised by the maximum value attained. All other parameters as in Table A4. Dynamics are shown for a single simulation.

**Supplementary Table 1**- The propensity functions for each of the event types in the population-level model.

**Supplementary Table 2**- The propensity functions for the various event types for the within-host model.

**Supplementary Table 3**- Default parameters for the between-host model.

**Supplementary Table 4**- Default parameters for the within-host model.
